# Brazilian guidelines for the pharmacological treatment of pulmonary hype rtension and the importance of Latin American guidance in pulmonary arterial hypertension

**DOI:** 10.36416/1806-3756/e20260325

**Published:** 2026-08-13

**Authors:** Tomas Pulido, Guillermo Bortman

**Affiliations:** 1. Instituto Nacional de Cardiología Ignacio Chávez, Ciudad de México, México; 2. Residencia en Cardiología, Universidad de Buenos Aires, Buenos Aires, Argentina.; 3. Sanatorio de la Trinidad Mitre y Sanatorio de la Trinidad Palermo, Buenos Aires, Argentina.

The new Brazilian guidelines for the pharmacological treatment of pulmonary hypertension (PH) represent a multidisciplinary effort in the regional context, firmly placing Latin America within contemporary pulmonary arterial hypertension (PAH) discourse. Rather than serving only as a national statement, these guidelines offer a model for how Latin American health systems can adapt high-quality evidence to local realities while remaining aligned with European and North American standards.^1^

These guidelines were developed using the Grading of Recommendations Assessment, Development, and Evaluation (GRADE) and Preferred Reporting Items for Systematic Reviews and Meta-Analyses (PRISMA) methods and feature four predefined PICO (Patients of interest, Intervention to be studied, Comparison of intervention, and Outcome of interest) questions and systematic reviews that inform evidence-to-decision frameworks. This aligns Brazilian PH recommendations with the rigorous methodology of the ESC/ERS guidelines^2^ and with recommendations from the 6th World Symposium on PH.^3^ The panel reviewed more than 1,600 articles for PICO 1 and more than 13,000 related to PH due to left heart disease, synthesizing this evidence into ranked recommendations and clearly identifying areas with weak or no evidence. Crucially, these guidelines integrate PICO-based statements with a narrative review that addresses general measures, risk assessment, and treatment strategies across PH groups, which is especially helpful in Latin America, where resource limitations often influence clinical choices.^4^

By aligning definitions and classification with the 2024 ERS statement- mean pulmonary artery pressure > 20 mmHg and pulmonary vascular resistance > 2 WU for precapillary PH-while tailoring therapeutic choices to Brazilian availability, the document connects global consensus with local feasibility.

The PAH (Group 1) section is particularly comprehensive. It starts with broad measures often overlooked in typical pharmacological talks , such as psychological support, vaccination, pulmonary rehabilitation, pregnancy advice, perioperative planning, and palliative care. These guidelines portray PAH as a complex, multisystem disease rather than merely a hemodynamic issue. The segment on pregnancy is especially pertinent for Latin America. Although outcomes have improved in high-income countries with i.v. prostanoids, maternal-fetal mortality remains alarmingly high in developing nations. Consequently, the guideline still considers pregnancy in PAH generally contraindicated, permitting only rare exceptions for patients who respond long-term to calcium-channel blockers or are considered truly low-risk by expert teams.

The pharmacotherapy section is based on solid evidence yet remains practical. It continues to focus on the three traditional pathways-endothelin, nitric oxide, and prostacyclin-while also including the newer activin/TGF-β pathway via sotatercept. Starting with dual oral therapy using an endothelin receptor antagonist (ERA) and a phosphodiesterase 5 inhibitor (PDE5i) is strongly advised, based on the AMBITION trial and extended to other ERA/PDE5i combinations available in Brazil.^5^ ^-^ ^7^ For patients who do not achieve low-risk status, sequential triple therapy with a prostacyclin analogue or selexipag is recommended, supported by randomized studies and aligned with international standards.^8^ Finally, for patients on combination therapy who remain at intermediate risk, the panel suggests switching a PDE5i to riociguat (conditional recommendation, very low quality evidence).^9^ Sotatercept is presented as a fourth pathway option for patients who remain non-low risk despite optimized vasodilator treatment, supported by data from the STELLAR trial and subsequent high-risk phase 3 results demonstrating significant improvements in outcomes.^10^ ^,^ ^11^

This step-by-step approach is outlined in a straightforward algorithm that uses risk assessment at baseline and every 3-4 months to determine when to escalate care, aiming to keep risk low. The authors categorize follow-up risk simply as “low” or “not low,” rather than using more detailed systems like COMPERA 2.0,^12^ a choice that better reflects the treatment options available in Brazil. The guideline also offers a multiparametric risk model aligned with European standards, including 6MWD, WHO functional class, natriuretic peptides, echocardiography, cardiopulmonary exercise testing, hemodynamics, and cardiac magnetic resonance (CMR), along with a minimal set of noninvasive assessments for routine follow-up.^2^ This tiered approach is especially useful in Latin America, where access to serial right heart catheterization or CMR outside major centers may be limited.

A notable strength of this document is its readiness to reject unproven treatments. In PH caused by left heart disease (Group 2) and lung disease/hypoxemia (Group 3), the panel carefully analyzes randomized data and largely advises against the off-label use of PAH-specific drugs that are often employed empirically. For Group 2 PH, sildenafil and other PDE5i are not recommended routinely because the evidence is inconsistent and may even be harmful; riociguat lacks enough evidence for a definitive recommendation; and bosentan and macitentan are discouraged, with macitentan strongly advised against due to minimal benefit and fluid retention risks.^13^ The key message is that treatment should focus on optimal heart failure and valvular therapy, rather than applying Group 1 drugs to different disease processes.

Group 3 PH guidelines remain cautious and nuanced. In COPD-related PH, evidence on PDE5i and tadalafil is conflicting and limited, so no clear recommendation is provided for or against PDE5i use; bosentan is advised against due to lack of benefit and possible harm. For PH caused by interstitial lung disease (ILD), riociguat is not recommended based on the RISE-IIP trial,^14^ and ambrisentan is also discouraged because it worsened clinical outcomes in idiopathic pulmonary fibrosis.^15^ Inhaled treprostinil shows benefits in ILD-PH, supported by the INCREASE trial, and is conditionally recommended as an adjunct in ILD treatment.^16^ The guideline also appropriately highlights the PERFECT study, which found increased adverse events with inhaled treprostinil in COPD-PH, and accordingly advises against its use in COPD.^17^

The chronic thromboembolic pulmonary hypertension (CTEPH) section highlights that pulmonary endarterectomy is the only potentially curative option. It also underscores a multimodal approach that includes lifelong anticoagulation, balloon pulmonary angioplasty, and riociguat for cases that are inoperable or have residual disease. Notably, the guideline points out that although riociguat has approval in Brazil, it is not accessible through the public healthcare system, exposing a gap between regulatory approval and actual availability. For Group 5 PH, characterized by heterogeneity and limited evidence, the main recommendation is to address the underlying cause and refer patients to specialized centers.

Beyond the scientific considerations, these Brazilian guidelines are significant because they represent a Latin American perspective on PH care. The document consistently mentions which medications are approved by the Brazilian Health Regulatory Agency and accessible through the Brazilian Unified Health Care System, noting that access to drugs such as macitentan, riociguat, or sotatercept may be limited outside supplementary health care. It also highlights the importance of specialized centers for managing PAH, CTEPH, and certain Group 3 and Group 5 patients, acknowledging the uneven distribution of expertise and resources nationwide. The guidelines openly address specific challenges such as high maternal-fetal mortality in PAH, difficulties in obtaining genetic testing, limited transplant options, and decisions influenced by resource availability for imaging and hemodynamics.

This Brazilian experience can serve as a model for the broader Latin American region. Many countries face similar epidemiological patterns, public-private sector fragmentation, and limitations in accessing prostanoids, soluble guanylate cyclase stimulators, and now sotatercept. The guideline’s strength is its refusal to blindly adopt European or North American algorithms without tailoring them to local contexts.

By identifying where evidence is strong and where it is weak, the guideline also defines a research agenda for Latin America. Key priorities include real-world outcome studies of risk-stratified algorithms in Brazilian and regional cohorts, cost-effectiveness and budget-impact analyses of dual therapy, triple therapy, and novel agents such as sotatercept, prospective work in schistosomiasis-associated PAH and PH-ILD in endemic connective tissue diseases, and implementation science focused on referral pathways to PH and CTEPH centers.

In summary, the updated Brazilian guidelines offer an evidence-based, practical, and context-sensitive approach to managing PH, benefiting not just Brazilian clinicians and patients but also the broader Latin American region. They demonstrate that applying strict GRADE methodology and clear, risk-focused algorithms can work alongside transparent acknowledgment of local resource limitations, resulting in a dynamic document that guides current care and informs future research.
